# Tumor angiogenesis targeting and imaging using gold nanoparticle probe with directly conjugated cyclic NGR†

**DOI:** 10.1039/c7ra10155d

**Published:** 2018-01-05

**Authors:** Minghao Wu, Yanyan Zhang, Ying Zhang, Mingjie Wu, Menglin Wu, Hongyi Wu, Lin Cao, Liang Li, Xue Li, Xuening Zhang

**Affiliations:** Department of Medical Imaging, Second Hospital of Tianjin Medical University Tianjin 300211 P. R. China luckyxn_tianjin@163.com lx_1229@163.com +86-22-88329407; Institut National de la Recherche Scientifique-Énergie Matériaux et Télécommunications Varennes Quebec Canada J3X 1S2

## Abstract

Angiogenesis is a vital process for the growth and metastasis of malignant tumor. Visualization of tumor angiogenesis is thus of great importance in the evaluation of biologic aggressiveness as well as monitoring of the response to anti-angiogenic therapy. Herein, we developed a probe based on gold nanoparticles (GNPs) directly surface-functionalized with a tumor-homing cyclized asparagine–glycine–arginine peptide (SH–cNGR) and carboxylpoly(ethylene glycol)thiol (SH–PEG–COOH) *via* Au–S bonds. The obtained GNPs–PEG@cNGR probe was used to target the aminopeptidase-N (APN/CD13), which overexpressed in the endothelium of tumor angiogenesis. The CD13 binding affinities of the peptides were assessed by a receptor binding assay based on HUVEC and HepG2 cell (*e.g.* fluorescence imaging and X-ray computed tomography (CT)). The tumor targeting efficacy and the distribution of the GNPs–PEG@cNGR *in vivo* were further evaluated in a subcutaneous 4T1 xenograft model by CT imaging and immunohistochemistry study. These results showed that the GNPs–PEG@cNGR rapidly and specifically bound to the tumor vasculature after intravenous injection. Quantitative studies demonstrated that GNPs–PEG@cNGR showed significantly higher and faster tumor uptake after intravenous injection compared to unlabeled GNPs–PEG. Moreover, the distribution of tumor enhancement was consistent with the spatial distribution of angiogenic blood. These results suggest that the designed GNPs–PEG@cNGR probe may serve, in principle, as a promising CT contrast agent for targeted angiogenesis imaging and quantitative analysis.

## Abstract 2

Angiogenesis is a vital process for the growth and metastasis of malignant tumor.

## Graphical abstract

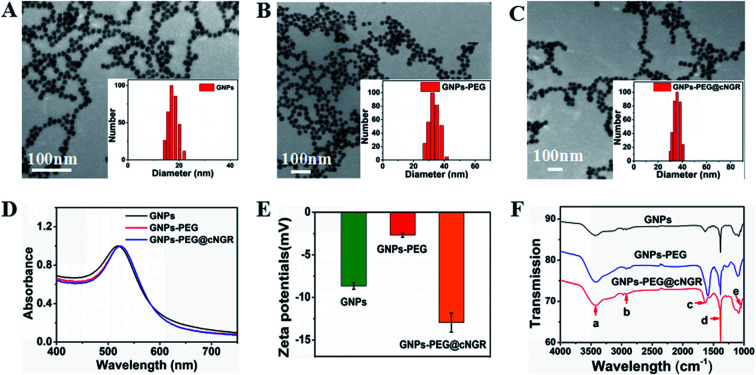

## 1 Introduction

Tumor angiogenesis has become an important predictor in cancer diagnosis and prognosis for its crucial role in tumor progression, particularly tumor growth, development, and metastasis.^1^ Assessment of tumor angiogenesis provides a gateway for precisely identifying tumor staging and invasion. However, most quantitative studies of angiogenesis in the clinic rely on the measurement of microvascular density (MVD) by calculating the maximal number of blood vessels per unit area of histological tissue section,^2,3^ which requires an intact tissue sample by invasive approaches. In addition, this method also has been proven unreliable in numerous tumors due to their high heterogeneity. Consequently, it is of great importance to quantitatively evaluate tumor vascularization *in vivo* by developing non-invasive and reliable imaging techniques.

Computed tomography (CT) is one of the most commonly used clinical diagnostic imaging techniques to visualize the major vascular because of their advantages of high temporal and spatial resolution, valuable 3-dimensional (3D) tomography information, and cost effectiveness.^4,5^ The application of CT imaging offers a promising approach for noninvasively evaluating tumor microvasculature. However, the conventionally used clinical CT contrast agents (*e.g.*, iodine) were often insufficient to visualize the tumor angiogenesis due to low sensitivity and the lack of specificity and limited retention.^6–8^ Hence, CT is not so widely considered for tumor angiogenesis imaging. Therefore, the development of excellent CT contrast agents to directly visualize tumor angiogenesis with long retention is necessary to advance the field of non-invasive tumor angiogenesis imaging.

Owing to their great X-ray attenuation property, enhanced permeability, and retention (EPR),^9–12^ colloidal gold nanoparticles (GNPs) have been intensively designed and fabricated *via* various methods. An ideal GNPs-based contrast agent for tumor angiogenesis should possess long circulation and vasculature targeting capacity.^13^ To address these challenges, polyethylene glycol (PEG) is often modified on the surface of GNPs and subsequently conjugated with neovasculature target ligands *via* covalent cross-linking.^14–19^ However, these bioconjugations (*e.g.* an amidation reaction) require a complicated multi-step synthesis and multifarious reagents, resulting in an aggregation of the obtained GNPs and poor deposition in tumor angiogenic site.^20^

**Scheme 1:**
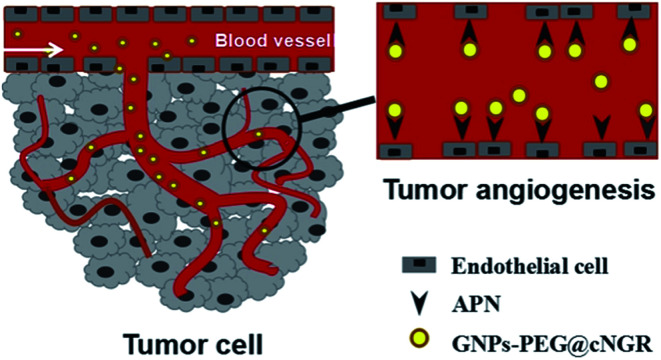
The targeting mechanism of the GNPs–PEG@cNGR probe to tumor angiogenesis.

**Scheme 2:**
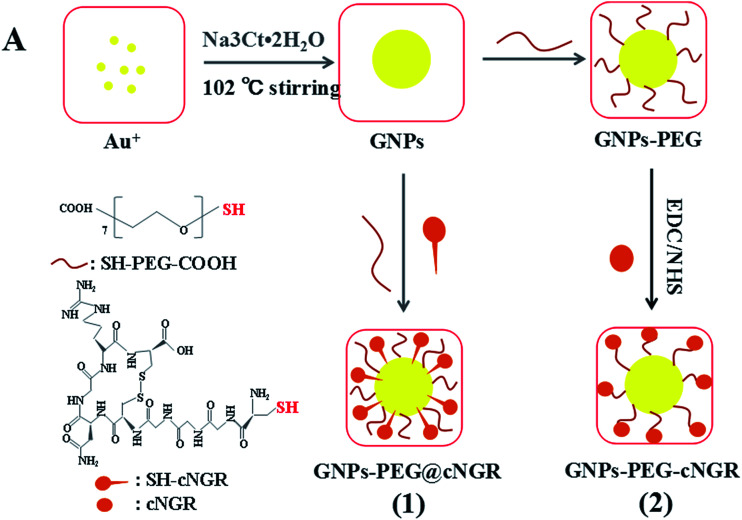
The comparison between (1) the present preparation method and (2) the traditional synthetic method for the GNPs–PEG@cNGR probe.

Herein, we strategically designed and synthesized a cNGR-conjugated and PEGylated GNPs (GNPs–PEG@cNGR) probe directly through the Au–S bonds (Scheme 1). The SH–cNGR with cysteines on the C terminal was designed to modify GNPs through the well-studied Au–S interaction. This modification method simplifies the synthetic steps, shortens the reaction time, and reduces the cost. As a result, a large volume of such a contrast agent can be easily produced by a very green process. The gold nanoparticles-probe functionalized by conjugating SH–cNGR as a CD13-specific targeting ligand could actively target tumor angiogenesis (Scheme 2). In this study, the particle size, morphology, and electric potential of GNPs–PEG@cNGR were comprehensively investigated. Then, the intracellular uptakes as well as the quantitative analysis were performed both in HUVEC and HepG2 cells. Moreover, the effectiveness of the probe as a CT contrast agent in 4T1 tumor-bearing Balb/c nude mice was further evaluated. Finally, immunohistochemistry and hematoxylin and eosin (H&E) staining studies were performed to explore tumor vascular distribution and biocompatibility. The obtained GNPs–PEG@cNGR probe exerted an excellent tumor angiogenesis target effect and a CT imaging potential, which had access to assessing the metastasis and malignancy of the tumor. To the best of our knowledge, this is the first report related to the development of SH–cNGR-tagged GNPs for *in vivo* CT imaging of tumor angiogenesis.

## 2 Experimental

### 2.1 Materials

Tetrachloroauric acid trihydrate (HAuCl_4_·3H_2_O), sodium citrate dihydrate (Na_3_Ct·2H_2_O), and carboxylpoly(ethylene glycol)thiol (SH–PEG–COOH) were obtained from Sigma-Aldrich (Louis, USA). Cyclic CYGGCNGRC (SH–cNGR) was purchased from GL Biochem Co. Ltd. (Shanghai, China). Dulbecco's modified eagle's medium (DMEM), trypsin–EDTA, penicillin–streptomycin, and fetal bovine serum (FBS) were purchased from Gibco-BRL (Grand Island, New York). 3-(4,5-Dimethylthiazol-2-yl)-2,5-diphenyltetrazolium bromide (MTT) was purchased from Merck (Germany). All other chemicals were of analytical grade and used without further purification.

The HepG2 cell line, HUVEC cell line, and 293T cell line were acquired from Molecular Imaging Lab of Tianjin Medical University, China. The female Balb/c mice were obtained from Institute of Radiology of Chinese Academy of Medical Sciences.

### 2.2 Synthesis of GNPs–PEG@cNGR

#### 2.2.1 Preparation of GNPs

GNPs of 13 nm diameter were synthesized according to a previous report.^21^ Initially, 150 mL of 2.2 mM Na_3_Ct·2H_2_O in deionized water was heated to 102 °C. Then, 1 mL of 24 mM HAuCl_4_·3H_2_O solution was quickly added to the Na_3_Ct solution under vigorous stirring at 102 °C. After 10 min, the color of the solution changed to wine red, followed by the formation of GNPs. Subsequently, the solution was allowed to cool down to room temperature with continuous stirring.

#### 2.2.2 Preparation of GNPs–PEG

To prepare PEGlyated gold nanoparticles (GNPs–PEG), an aqueous solution of SH–PEG–COOH (1 mL, 4 mg mL^−1^) was added to the GNPs solution to obtain GNPs–PEG and then stirred at room temperature overnight to allow complete exchange of the citrate with thiol. Next, the mixture was purified by centrifugation (13 000 rpm for 30 min at 4 °C) twice. After the supernatant was removed, the pellet was redispersed in phosphate buffer (PBS, pH 8).

#### 2.2.3 Preparation of GNPs–PEG@cNGR probe

To prepare cNGR/PEG-modified GNPs, gold nanoparticles bearing SH–PEG–COOH and SH–cNGR were prepared by simultaneously incubating GNPs with the solution of SH–cNGR and SH–PEG–COOH. To this end, the pH of the GNPs solution was adjusted to 9 with 1 M sodium hydroxide (NaOH) buffer (150 : 1 volume ratio). Subsequently, 1 mL of the mixture of SH–PEG–COOH (2 mg) and SH–cNGR (220 μg) dispersed in deionized H_2_O was poured into GNPs. The mixture was incubated for 8 h at room temperature under gentle stirring. The obtained solution was then purified by a series of centrifugation (13 000 rpm for 30 min each at 4 °C). The supernatant was removed and the pellet, obtained as the final product, was purified using size exclusion filters with a 10 kDa molecular weight cutoff (Millipore, Amicon Ultra-0.5) using PBS as an eluent, followed by the formation of the GNPs–PEG@cNGR probe.

#### 2.2.4 Preparation of FITC coupled GNPs–PEG@cNGR and GNPs–PEG

To prepare FITC coupled GNPs–PEG@cNGR and GNPs–PEG, 1 mg of the GNPs–PEG@cNGR probe, previously synthesized, was dispersed into 1 mL crosslinking reaction solution containing 7.56 mg Na_2_CO_3_, 1.06 mg NaHCO_3_, and 7.36 mg NaCl, sonicated for 2 min, and stored at 4 °C for 10 min. Subsequently, 0.3 mg FITC in 300 μL of DMSO was poured into the above solution under gentle and continuous stirring and incubated in the dark for 8 h at 4 °C. Next, 20 μL NH_4_Cl solution (5 M) was poured into the above solution under gentle and continuous stirring to terminate the above reaction and then incubated in the dark for 2 h at 4 °C. The final GNPs–PEG@cNGR@FITC was retrieved by ultrafiltration with a Millipore centrifugal filter unit (Amicon Ultra-0.5; MWCO 100 000), washed three times, and resuspended in 0.5 mL of PBS (pH 7.4).

In addition, FITC-conjugated GNPs–PEG (GNPs–PEG@FITC) was also prepared as a control. Briefly, the preparation was similar to the fabrication of GNPs–PEG@cNGR, but 220 μL SH–cNGR (1 mg mL^−1^) was replaced by 15 μL 2-aminoethanethiol (20 mM).

### 2.3 Characterizations of the probe

The size, morphology, and nanostructure of the GNPs, GNPs–PEG, and GNPs–PEG@cNGR were characterized using a TEM (JEM-2010HR, JEOL, Japan) operated at an operating voltage of 200 kV by pouring the probe solution onto a carbon-coated copper grid and allowing it to dry at room temperature. A UV 3150 spectrophotometer (Shimadzu, Japan) was used to analyze the maximum absorption wavelength of the probe using quartz cuvettes with an optical path of 1 cm. The surface compositions of the probe were investigated by Fourier transform infrared (FTIR) spectra of the GNPs, GNPs–PEG, and GNPs–PEG@cNGR. The FTIR spectra were recorded on a Nicolet Avatar FTIR model 330 spectrometer (Thermo, America) in the wavenumber range from 500 to 3800 cm^−1^. Dynamic light scattering (DLS) measurements of the GNPs, GNPs–PEG, and GNPs–PEG@cNGR were performed by photon correlation spectroscopy using a Malvern Zeta sizer 3000 HS (Malvern, UK) equipped with a standard 633 nm-laser at 9° inclination. The surface *ζ*-potential charges of the GNPs, GNPs–PEG, and GNPs–PEG@cNGR were determined with the same instrument equipped with an AQ-827 electrode. The data were calculated automatically from electrophoretic mobility based on the Smoluchowski theory.

### 2.4 X-ray attenuation measurements of the probe

The aqueous solutions of GNPs–PEG@cNGR and iohexol with different Au or iodine concentrations were prepared in 1.5 mL Eppendorf tubes and placed in a self-designed scanning holder. CT scans were performed on a GE Light Speed VCT imaging system (GE Medical Systems) at 100 kV, 80 mA, and a slice thickness of 0.625 mm. Contrast enhancement was determined in the Hounsfield units (HU) for each concentration of GNPs–PEG@cNGR or iohexol.

### 2.5 Stability of the probe

The stability of the GNPs–PEG@cNGR probe was estimated by a UV 3150 spectrophotometer and a Malvern Zeta sizer 3000 HS (Malvern, UK). The probe was incubated in deionized water and DMEM with 10% (v/v) fetal bovine serum (FBS, Gibco) for four months at room temperature. During this period, the UV-vis spectra and the solution situation of GNPs–PEG@cNGR were monitored constantly. Simultaneously, the hydrodynamic size distribution was measured at 0 and 120 days.

### 2.6 Cytotoxicity of the probe *in vitro*

For further biomedical application, the cytotoxicity of the GNPs–PEG and GNPs–PEG@cNGR probe was evaluated by the typical MTT reduction assays, which were based on the mitochondrial conversion of the tetrazolium salt into a dye with the absorption in the visible region. For this purpose, the HepG2 cell line (human hepatocellular carcinoma cell line, high level of expression of APN^22^), HUVEC cell line (human umbilical vein endothelial cells, high level of expression of APN^23^), and 293T cell line (human renal epithelial cell line) were cultured in DMEM, which were supplemented with 10% FBS and 1% penicillin/streptomycin at 37 °C in a humidified atmosphere containing 5% CO_2_. Then, the cells were seeded in a 96-well microculture plate with 5 × 10^4^ cells per well and incubated with the GNPs–PEG and GNPs–PEG@cNGR probe at different concentrations of the particles (18.75, 37.5, 75, 150, and 300 μg of Au per mL) and PBS as a negative control. The cell viability was assessed after 12 h incubation at 37 °C. Subsequently, 10 μL of the MTT solution was added. After co-incubation for another 4 h, the entire medium was removed, followed by the addition of 100 μL dimethyl sulfoxide/well and incubation for 30 min at 37 °C to dissolve formazan. The OD values at 490 nm were measured with a microplate reader (Victor X5, PerkinElmer). Finally, to compare the relative viability, five wells of each concentration were set up and all of the data were presented as the mean percentage ±SD. The cell viability was calculated as follows:Cell viability (%) = OD_net490 (sample)_/OD_net490 (control)_ × 100%where OD_net490 (sample)_ is the absorbance at 490 nm of the cells incubated with the GNPs–PEG and GNPs–PEG@cNGR probe and OD_net490 (control)_ is the absorbance at 490 nm of the cells incubated with the PBS.

### 2.7 Specificity of the probe

To investigate whether the GNPs–PEG@cNGR probe could specifically target a CD13 receptor, the cell fluorescence/CT imaging *in vitro* was performed. For fluorescence imaging, following incubation of the HUVEC cells seeded into the confocal dishes at 37 °C for 24 h, the cells were then incubated with GNP–PEG@FITC, GNP–PEG@cNGR@FITC, and GNPs–PEG@cNGR in the presence of excess free SH–cNGR at a concentration of 300 μg mL^−1^ for another 4 h at 37 °C. The cells treated with PBS acted as a control group. After being washed three times with PBS, the cells were fixed by polyparaformaldehyde for 20 min, followed by staining with 4′,6-diamidino-2-phenylindole (DAPI) for 15 min. Finally, the cells were examined by a TCS SP2 confocal microscope (Leica, Germany) with 494 nm laser-excitation for FITC and 364 nm laser excitation for DAPI. The nuclei stained with DAPI were blue and the FITC-modified GNPs were green. For the cell CT imaging, the HepG2 cells were seeded in the culture plates with a density of 1 × 10^6^ per plate. After being cultured for 24 h, the cells were co-cultured with GNPs–PEG, GNPs–PEG@cNGR, and GNPs–PEG@cNGR plus excess free SH–cNGR at 300 μg mL^−1^ for 4 h. Then, the cells were rinsed three times with PBS to remove free particles, collected and counted. The cells were resuspended in 100 μL PBS in 0.5 mL Eppendorf tubes. Each tube contained 2.0 × 10^6^ cells. These tubes were placed in a self-designed scanning holder. The CT images and the corresponding attenuation measurements were acquired using a clinical CT imaging system at 120 kV and 200 mA. After being scanned, the resuspended cells were centrifuged again and treated with successively adding 1.0 mL of aqua regia, followed by sonication for 20 min in a hot water bath to completely digest the cells and dissolve the GNPs. The resultant solution was diluted with 4 mL of deionized water, followed by ICP-MS (DRCII, PerkinElmer) analysis to determine the gold element contents in the cells, which were expressed in picogram (pg) of iron per cell.

### 2.8 CT imaging *in vivo*

All animal experiments were approved by the institutional committee for animal care and the National Ministry of Health and performed in accordance with the procedures of Institutional Animal Care and Use Committee for animal treatment under sterile conditions. In the right flank of female Balb/c mice (6 weeks old, average body weight: 22 g), the 4T1 cells were implanted with 2 × 10^6^ in PBS (60 μL) subcutaneously. When the tumors reached 200–400 mm^3^, the mice received 0.1 mL of GNPs–PEG or GNPs–PEG@cNGR (10 mg mL^−1^) through intravenous injection. The images of the mice were acquired from a GE Light Speed VCT clinical imaging system (USA) at 1 h, 2 h, 4 h, 8 h, 12 h, and 24 h post-injection under the same CT scanning parameters as with *in vitro* attenuation measurements. In addition, *in vivo* safety was evaluated. Here, GNPs–PEG and PBS were included as the positive and negative controls, respectively.

### 2.9 Immunohistochemical and histochemical studies

After CT imaging, the mice were sacrificed under anesthetic conditions. Tissues of interest (heart, liver, spleen, lung, and kidney) and the tumors were excised and preserved in a 10% formalin solution for two weeks. Hematoxylin and eosin (H&E) staining was performed on the main organs to evaluate the safety of the GNPs–PEG@cNGR probe to the organs and immunohistochemical staining was performed on the obtained tumors against CD31 for analyzing the distribution of tumor angiogenesis. For H&E staining, formalin fixed tissues from each organ were embedded into paraffin and then the paraffin-embedded tissues were sectioned into 4 mm thickness. Each histological section was documented by a dissecting microscope. For immunohistochemical staining, the tumors were embedded in paraffin and then sectioned. The tumor slices (10 μm thick) were deparaffinized in xylene, dehydrated in the solutions of graded ethanol, and then incubated in hydrogen peroxide (3%) for 10 min. After antigen retrieval was performed by a microwave treatment, the sections were first incubated with anti-CD31 antibody at 4 °C overnight and then incubated for 1 h with a biotinylated goat antirat IgG. Finally, the tumor sections were counterstained using hematoxylin and returned to the ammonia solution. MVD was assessed according to the international consensus report. The analysis of immunohistochemical staining was performed using a light microscope. To quantify tumor microvessel density (MVD), six immuno-stained slides (three from the periphery and three from the core) were investigated first at 100× magnification (FOV = 500 × 350 mm) to identify these areas with the highest number of vessels (so-called ‘hot spots’) from three representative sections per tumor. Then, counts were performed on 10 fields in the hot spot at 400× magnification by two independent investigators who were blinded to clinical features and survival data and the mean was calculated.

### 2.10 Pharmacokinetics and biodistribution studies

ROIs defining the spleen, liver, kidney, heart, aorta, and bladder were drawn manually in the CT imaging system. The CT value was obtained at pre-injection and 1 h, 2 h, 4 h, 8 h, and 24 h post-injection. The CT density was averaged over the entire tissue.

### 2.11 Statistical analyses

All results presented are the means or means ± standard deviation (SD) of the experiments repeated at least three times. A Student's *t*-test was used to evaluate the significance among different groups. A *p* value of less than 0.05 was considered to be significant.

## 3 Results and discussion

### 3.1 Preparation and characterization of the probe

**Fig. 1:**
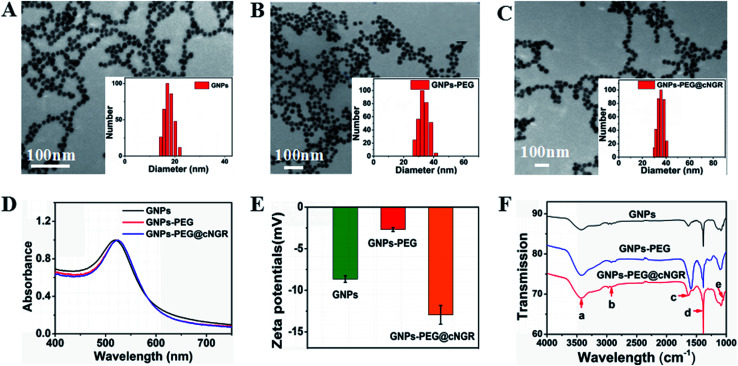
Basic characterization of the probe. The TEM images show (A) GNPs, (B) GNPs–PEG, and (C) GNPs–PEG@cNGR. The 100 nm scale bar applies to all images. The insets: the histograms for hydration kinetic diameter size distribution. (D) UV-visible absorption spectra of the as-prepared GNPs, GNPs–PEG, and GNPs–PEG@cNGR. (E) Zeta potentials of GNPs, GNPs–PEG, and GNPs–PEG@cNGR. (F) The ATR-FTIR spectra of GNPs, GNPs–PEG, and GNPs–PEG@cNGR. (a) 3430 cm^−1^ OH stretching H-bonded, (b) 2920–2855 cm^−1^ CH_2_ stretching, (c) 1631 cm^−1^ amide I, (d) 1384 cm^−1^ amide III (C–N stretch vibration), and (e) 1087 cm^−1^ COC stretching.

In the present study, we prepared a GNPs–PEG@cNGR using a one-step functionalized method. As shown in Scheme 2, SH–cNGR and SH–PEG–COOH were conjugated on the surface of the GNPs *via* the Au–S bonds. Compared to the traditional targeted-peptide modified methods, our synthetic method simplifies the synthetic steps, shortens the reaction time, and reduces the cost. As a result, a large volume of such GNPs–PEG@cNGR probes can be easily produced by a very green process. As shown in the TEM images in Fig. 1A–C, the GNPs, GNPs–PEG, and GNPs–PEG@cNGR possessed a uniform spherical morphology. The corresponding histograms of the hydration kinetic diameter size distribution (insets) of nanoparticles revealed that the GNPs, GNPs–PEG, and GNPs–PEG@cNGR had a spherical shape with a mean diameter of 17.0, 32.3, and 35.7 nm, respectively, with a quite narrow size distribution. It was observed that no visible changes such as morphology and size between GNPs–PEG and GNPs–PEG@cNGR appeared, which confirmed that no aggregation occurred after the conjugation of SH–PEG–COOH and SH–cNGR with GNPs. The surface plasmon absorption of the GNPs, GNPs–PEG, and GNPs–PEG@cNGR in distilled water appeared at a wavelength of 519.5, 522, and 525 nm, respectively (Fig. 1D). The slight red-shift from 519.5 to 525 nm, which might attribute to the polymer layer that changed the refractive index around GNPs, indicated a successful surface modification of GNPs.^24^ It has also been generally reported that the peak position of the surface plasmon absorption depended on the size and shape of GNPs.^25^ The surface zeta potential of the GNPs, GNPs–PEG, and GNPs–PEG@cNGR, as shown in Fig. 1E, were −8 ± 0.40 mV, −2.7 ± 0.23 mV, and −13 ± 1.12 mV in pure water, respectively. The surface potential of GNPs–PEG was lower than that of GNPs, which might be due to the fact that the charge strength of carboxyl groups of PEG was weaker than that of citrate on the surface of GNPs. The increase of the surface negative potential of the obtained GNPs–PEG@cNGR after conjugation with SH–cNGR can be explained by the fact that the terminal carboxyl group of SH–cNGR and SH–PEG–COOH on the surface of GNPs in an alkaline condition are directed into the surrounding solution to keep the stability of the colloidal solution. To further verify the attachment of SH–PEG–COOH and SH–cNGR, the GNPs, GNPs–PEG, and GNPs–PEG@cNGR were characterized using Fourier transform infrared spectroscopy (FTIR). As shown in Fig. 1F, the GNPs–PEG and GNPs–PEG@cNGR showed OH stretching (3430 cm^−1^), CH_2_ stretching (2920–2855 cm^−1^), and COC stretching (1087 cm^−1^) bands in common. These results indicated that SH–PEG–COOH was successfully immobilized on the surface of gold nanoparticles. Moreover, amide I bond (1631 cm^−1^, carbonyl stretch vibration) and amide III bond (1384 cm^−1^, C–N stretch vibration) only appeared in GNPs–PEG@cNGR, which illustrated the successful conjunction of SH–cNGR on the surface of GNPs.

### 3.2 X-ray attenuation property of the probe

**Fig. 2:**
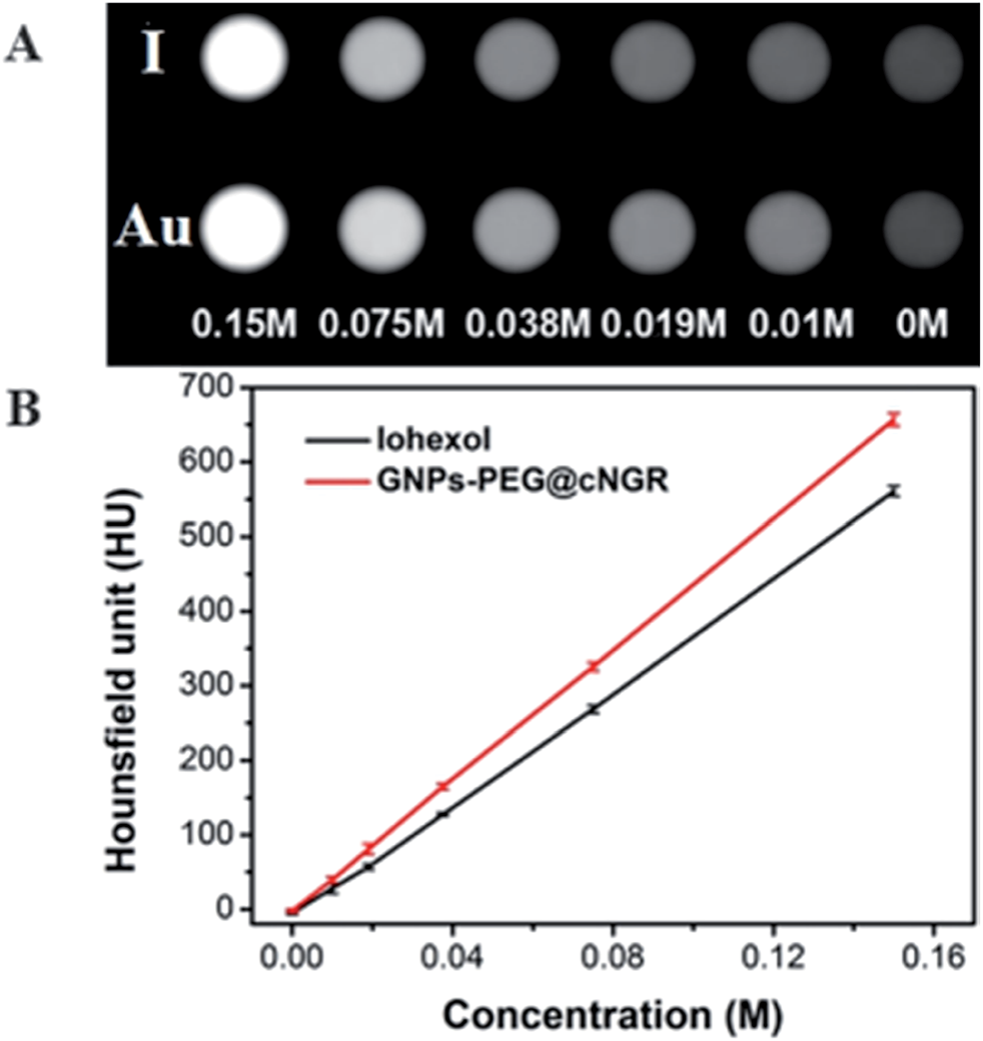
(A) Transverse CT images and (B) X-ray attenuation intensity (HU) of GNPs–PEG@cNGR and iohexol as a function of the molar concentration of the radiodense element (Au or I).

To examine the feasibility of the developed GNPs–PEG@cNGR in the CT imaging applications, the X-ray attenuation property of the probe was compared with iohexol (Fig. 2A). It was found that with an increase in the concentration of GNPs–PEG@cNGR or iohexol, the brightness of the GNPs–PEG@cNGR probe was more prominent than that of iohexol at the same concentration of the radiodense element (Au or I), particularly at high concentrations. Quantitative analysis (Fig. 2B) showed that the Hounsfield unit of the GNPs–PEG@cNGR was higher than that of iohexol at the same concentration and increased linearly with the concentration. These results suggested that the prepared GNPs–PEG@cNGR had a great potential for the application in CT imaging. The cause of this phenomenon may be due to the higher atomic number of Au than that of iodine, which resulted in heavier X-attenuation of GNPs–PEG@cNGR.

### 3.3 Stability of the probe

The colloidal stability of nanoparticles can greatly affect their biomedical applications. Since the aggregation behavior of nanoparticles can be directly reflected by the change of their absorption characteristics in the UV-vis spectrum^26^ and the change of hydrodynamic size, the colloidal stability of GNPs–PEG@cNGR at different time points was characterized by UV-vis light spectroscopy and DLS. As shown in Fig. S1,† no significant changes appeared in the absorption value and the absorption peak position and width. In addition, the solution color had no significant change compared with that of the foremost nanoprobe after four months and no flocculation and/or sedimentation appeared (Fig. S1† inset). The DLS results (Fig. S1†) showed that the hydrodynamic size distribution of the GNPs–PEG@cNGR did not change significantly with an increase in the incubation time. These results demonstrated that the synthesized GNPs–PEG@cNGR probe had a good colloidal stability in physiological conditions, which was essential for its further biomedical applications.

### 3.4 Cytotoxicity assessments of the probe

**Fig. 3:**
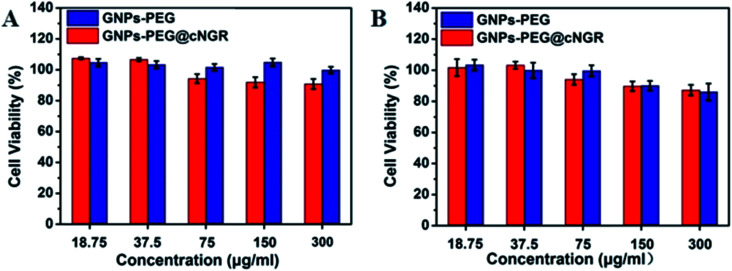
MTT viability assay of (A) HUVEC and (B) HepG2 cells after the treatment with GNPs–PEG@cNGR and GNPs–PEG at different concentrations from 18.75 to 300 μg mL^−1^ for 24 h.

Safety of nanoparticles was a critical parameter to determine their potential for further translational development. To assess their toxicity for the CD13 positive cell, we chose the HUVEC and HepG2 cell lines. Compared to that of the control group cells treated with PBS, the viability of both the HUVEC and HepG2 cell lines remained over 85% after incubating with either GNPs–PEG@cNGR or GNPs–PEG at relatively higher concentrations (300 μg mL^−1^) for 24 h (Fig. 3). Due to the excretion of GNPs–PEG@cNGR and GNPs–PEG primarily by the kidney (Fig. S4†), we also chose the 293T cell line to further confirm their cytotoxicity to the kidney. The results showed that at relatively higher concentrations (300 μg mL^−1^), the cell viability was still greater than 85% (Fig. S3†). These results confirmed that both types of GNPs had no visible toxic effect on not only the tumor cells, but also the microvasculature and renal epithelial cells. Therefore, both types of GNPs had a good biocompatibility and are suitable for biomedical applications.

### 3.5 Specificity of the probe

**Fig. 4:**
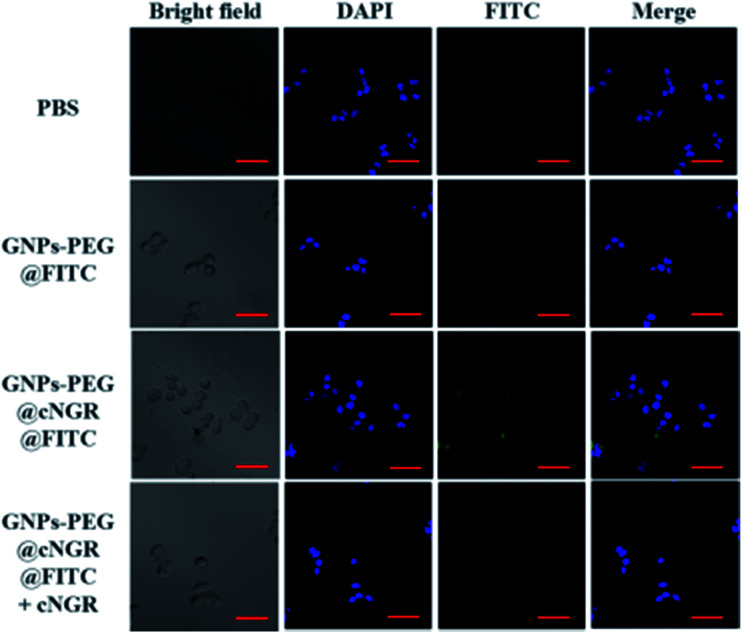
Specific uptake of GNPs–PEG@cNGR by the HUVEC cells. The confocal microscopic images of the HUVEC cells incubated with PBS (control group), GNPs–PEG@FITC, GNPs–PEG@cNGR@FITC, and GNPs–PEG@cNGR@FITC/SH–cNGR at a concentration of 300 μg Au per mL for 4 h. Scale bar: 50 μm.

To validate the practicability of GNPs–PEG@cNGR for tumor angiogenesis targeting, the cellular uptake of GNPs–PEG@cNGR by the CD13 positive HUVEC cells was investigated by tracking FITC with laser scanning confocal microscopy (LSCM) imaging. The HUVEC cells, a CD13 receptor-overexpressing umbilical vein endothelial cell line, could be specifically targeted by cNGR due to the specific recognition between cNGR and CD13 receptor. Since there were no targets available for GNPs–PEG, the GNPs–PEG would not be specifically internalized. When the CD13 receptors on the cells were blocked by excess free SH–cNGR, the GNPs–PEG@cNGR would also not be specifically internalized. As shown in Fig. 4, the GNPs–PEG@cNGR@FITC-treated group exhibited significantly strong green fluorescence signals, which suggested that SH–cNGR-modified GNPs could be effectively internalized into the HUVEC cells resulting in high efficient intracellular accumulation of GNPs–PEG@cNGR. In contrast, following the 4 h incubation period, there were no apparent green fluorescence signals in the HUVEC cells treated with the equivalent concentration of GNPs–PEG@FITC due to the lack of targeting peptide SH–cNGR. Furthermore, a competition experiment comparing the uptake efficacy of GNPs–PEG@cNGR in the presence of free SH–cNGR (referred to GNPs–PEG@cNGR@FITC/SH–cNGR) was performed to confirm that SH–cNGR-modified nanocomposites engaged in specific interactions with HepG2 *via* the recognition between the ligand (SH–cNGR) and the receptor (CD13). The obtained results showed that similar to the GNPs–PEG@FITC, there was no apparent green fluorescence in the HepG2 cells treated with GNPs–PEG@cNGR@FITC/SH–cNGR, which is attributed to the fact that the CD13 receptors were blocked by excess SH–cNGR. These results have confirmed to some extent that the GNPs–PEG@cNGR probe could specifically target the CD13 positive cells and the cellular uptake of the probe was primarily mediated by the CD13 receptor. Collectively, these observations demonstrated the potential of the linked SH–cNGR moieties in targeting overexpressing CD13 of tumor angiogenesis *via* a receptor-mediated pathway.

**Fig. 5:**
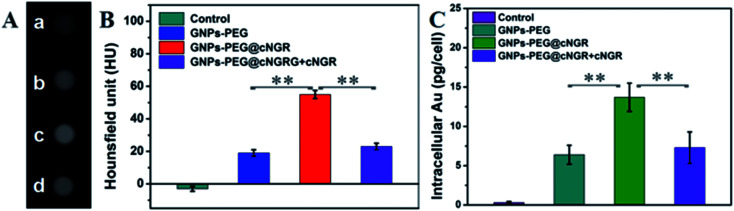
(A) Transverse micro-CT images and (B) CT values of the HepG2 cells incubated with PBS (a), GNPs–PEG (b), GNPs–PEG@cNGR (c), and GNPs–PEG@cNGR/SH–cNGR (d) at a concentration of 300 μg mL^−1^ for 4 h. (C) Intracellular Au contents quantified by ICP-MS. Data are presented as the means ± SD (*n* = 3). **, *p* < 0.01.

With the good targeting specificity of the developed GNPs–PEG@cNGR, we next explored the potential of the GNPs–PEG@cNGR for targeted CT imaging of the CD13 positive cells *in vitro*. Because the cells-CT imaging needs a huge number of cells to obtain a relatively precise result, we chose the HepG2 cells, which were easy to cultivate and have a short growth cycle rather than HUVEC. After incubation with the targeted GNPs–PEG@cNGR or the non-targeted GNPs–PEG for 4 h, the CT imaging of the HepG2 cells was performed (Fig. 5A). Given the fact that it is difficult to visually differentiate the brightness of the CT images of the cells treated with GNPs–PEG and GNPs–PEG@cNGR at a concentration of 300 μg mL^−1^, it is essential to perform a quantitative analysis of the CT signal intensity *via* the manufacturer's standard display program. As shown in Fig. 5B, the HepG2 cells incubated with the GNPs–PEG@cNGR probe showed significantly higher X-ray attenuation than those treated with the GNPs–PEG and GNPs–PEG@cNGR/SH–cNGR probes (*p* < 0.01). Furthermore, by quantifying the intracellular Au contents using ICP-MS, we also found that the cellular uptake of the GNPs–PEG@cNGR probe was indeed more than that of GNPs–PEG probe (*p* < 0.01) and free SH–cNGR peptide could inhibit the uptake of GNPs–PEG@cNGR probe (*p* < 0.01) (Fig. 5C). These results were well-consistent with the confocal microscopic results, which confirmed that the developed GNPs–PEG@cNGR probe enabled to target the CD13-overexpressing cells by the interaction between SH–cNGR and the CD13 receptors.

### 3.6 CT imaging *in vivo*

**Fig. 6:**
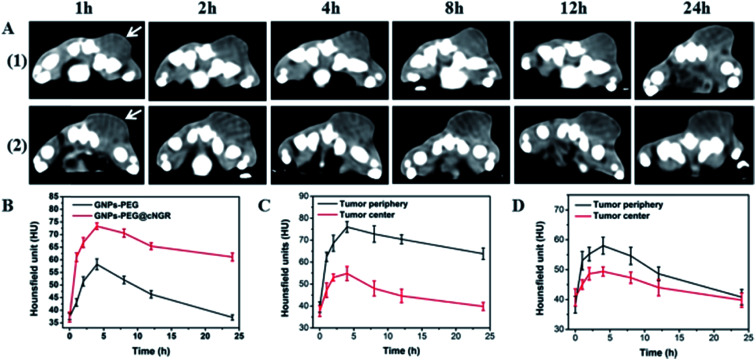
(A) Real-time *in vivo* CT imaging of the 4T1 tumor in the Balb/c mice after intravenous injection with (1) GNPs–PEG and (2) GNPs–PEG@NGR at the dose of 200 μL of 20 mg mL^−1^ at different time points and (B) the corresponding CT values. The white arrows indicate the local region of the tumor. The relationship between periphery intensity and central intensity of the tumor treated by (C) GNPs–PEG@cNGR or (D) GNPs–PEG.

**Fig. 7:**
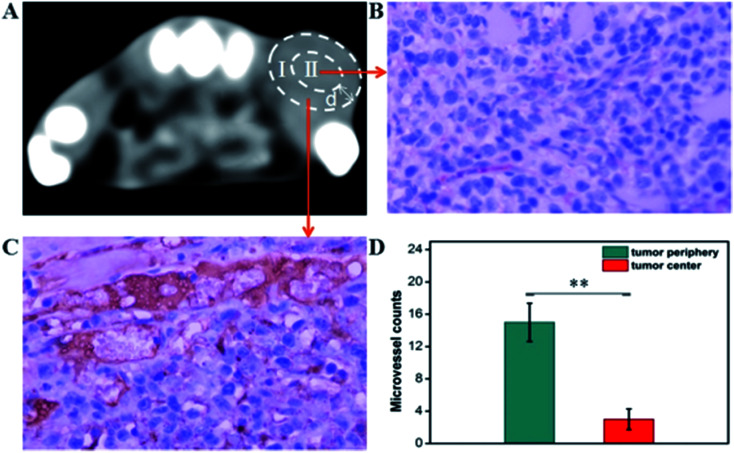
Microvessel density (MVD) in the xenograft 4T1 tumors. (A) *In vivo* tumor CT imaging. The representative ROIs of (I) tumor periphery and (II) tumor center. The representative micrographs of immunohistochemical detection of CD31-positive microvessel in the xenograft 4T1 tumors from (B) tumor center and (C) tumor periphery. (Final magnification, 400×). (D) Mean CD31-positive microvessel counts in the xenograft 4T1 tumors. The data are presented as the means ± SD (*n* = 5). **, *p* < 0.01, group(I) *vs.* group(II).

The CT imaging combined functionalized probe, which can specifically target the angiogenesis endothelial cells, can yield reference information for clinical assessment for tumor metastatic potential and prognosis. Encouraged by the above excellent performance of the developed GNPs–PEG@cNGR probe for targeted imaging of the CD13 positive cells *in vitro*, the potential of the *in vivo* tumor angiogenesis CT imaging was further explored on the 4T1 xenograft model (Fig. 6). As shown in Fig. 6A, compared to those of the tumors of the mice injected with the GNPs–PEG, the tumor areas post-injected with GNPs–PEG@cNGR display a faster and higher enhancement in the CT contrast. In addition, the CT values of the tumors treated with GNPs–PEG@cNGR were much higher than those treated with the non-targeted GNPs–PEG at the same time points (Fig. 6B). Specifically, dynamic contrast enhancement of neovascularity in the animals receiving CD13-targeted gold nanoparticles increased by 64.9% at 1 h and continued to improve through 4 h (96.5%), followed by only a slight drop at 8 h (90%). However, real-time contrast enhancement in the animals receiving non-targeted gold nanoparticles was about two folds less than that in the targeted group during the 24 h imaging period. These results indicated that the developed GNPs–PEG@cNGR probe can specifically target tumor angiogenesis, resulting in enhanced tumor angiogenesis CT imaging. To acquire the knowledge of the probe distribution in the tumors, each tumor was divided into a periphery (area I) and center (area II) (Fig. 7A). The tumor periphery was arbitrarily defined as the area that was obtained by eroding the distance (*d*) from the tumor border. The distance *d* (white arrows) corresponds to 25% of the tumor radius that was obtained if the tumor with volume V was an ideal sphere. For each animal, the distributed variable was calculated using the mean CT values (HU) from an ROI within the tumor. The mean CT values were obtained from each tumor ROI (center and periphery). As shown in Fig. 6C, the SH–cNGR-modified GNPs accumulated more in the periphery of the tumor than in the center after intravenous injection, particularly at 4 h. The difference was more distinct than that of the nonspecific GNPs–PEG. This phenomenon might be primarily attributed to the fact that the distribution of tumor angiogenesis was primarily concentrated on the periphery of the tumor.^27,28^ Moreover, the nonspecific GNPs–PEG also accumulated in the location of the tumor, but showed a relatively diffused distribution pattern (Fig. 6D), which might be attributed to endothelial permeability and retention effect (EPR) and “nanoparticle-induced endothelial leakiness” (NanoEL). The obtained GNPs within the range of about 15 nm are good NanoEL inducing particles; moreover, the human mammary endothelial cells are sensitive to Au induced NanoEL.^29^

### 3.7 Immunohistochemiscal study

To investigate the relationship between the distributions of tumor angiogenesis and intratumoral CT enhancement region, confocal immunofluorescence microscopy was used to examine the pathological section of the subcutaneous 4T1 tumors excised from the mice that had been imaged. As shown in Fig. 7, the majority of immunohistochemical staining of CD31 (*i.e.*, a general endothelial marker) was observed heterogeneously along the periphery of the tumors, which was consistent across all experimental groups. This result suggested the endothelial cell location and angiogenesis CD13 expression along the periphery of the 4T1 tumors. As such, the neovascular spatial distribution for the tumor was consistent with the regional contrast enhancement patterns of tumor CT imaging after injecting with the GNPs–PEG@cNGR probe (Fig. 6C). These results confirmed the active targeting effect of the GNPs–PEG@cNGR probe to CD13 over-expression tumor angiogenesis and the feasibility of the probe to quantitative evaluating tumor angiogenesis *in vivo*.

### 3.8 Pharmacokinetics and biodistribution *in vivo*

In order to track the *in vivo* pharmacokinetics and biodistribution of GNPs–PEG@cNGR, the CT values were employed from blood and major organs in real-time. As shown in Fig. S4,† no visible differences were found between the mice receiving GNPs–PEG@cNGR and GNPs–PEG treatment. It was clear that the strong enhancements in the bladders region were presented at 1 h post-injection. Moreover, a large quantity of GNPs–PEG@cNGR and GNPs–PEG accumulated in the organs of the reticuloendothelial system, such as the liver and the spleen. This finding demonstrated that besides tumor accumulation, GNPs–PEG@cNGR and GNPs–PEG can be excreted primarily through the renal-urinary routes and RES. In addition, at 24 h post-injection, the CT value restored to the original level, which revealed their excellent pharmacokinetics.

### 3.9 Histochemical study

**Fig. 8:**
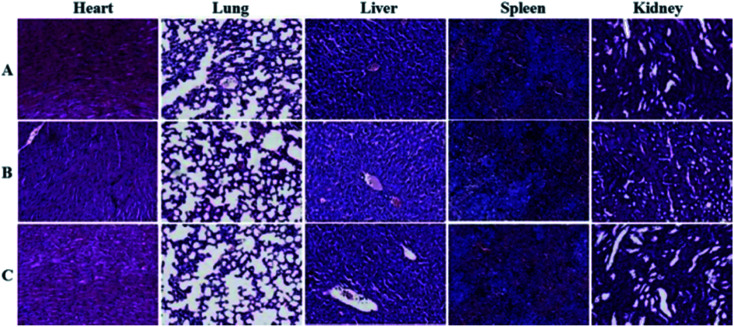
Histological analyses of main organs from the mice injected with 100 μL of (A) saline, (B) GNPs–PEG (20 mg mL^−1^), or (C) GNPs–PEG@NGR (20 mg mL^−1^).

The toxic side effects have always been of great concern in the development of nanomedicine. The safety effect of the agents on the major organs from mice was further assessed by histochemical analysis. Just as the indication from Fig. 8, by comparing histological results of the major organs of the mice injected with the GNPs–PEG@cNGR to those from the mice injected with PBS, we cannot find detectable tissue damage or other lesions such as necrosis, inflammatory, or pulmonary fibrosis. Collectively, these results illustrated that the GNPs–PEG@cNGR probe was biocompatible and was suitable for biomedical applications.

## 4 Conclusion

In summary, we successfully fabricated a novel nanoprobe GNPs–PEG@cNGR that targeted tumor angiogenesis to assess the malignant potential and therapeutic effect of breast cancer. SH–cNGR and SH–PEG were conjugated with GNPs directly through the Au–S bonds without any amidation reaction or bifunctional cross-linker. This coupling method was very simple and straightforward, resulting in large production, relative stability, and green synthesis. We found that the modified product GNPs–PEG@cNGR exhibited an excellent *in vitro* stability and desirable biocompatibility. Moreover, the SH–cNGR functionalized gold nanoparticles exhibited high specificity for the cells overexpressing CD13, enabling higher accumulation of GNPs by the CD13 positive cells *in vitro*. In particular, the GNPs–PEG@cNGR probe not only specifically target tumor angiogenesis and show an excellent CT imaging potential, but also can quantitatively evaluate tumor angiogenesis *in vivo*. The outstanding targeted tumor angiogenesis CT imaging performance of the obtained GNPs–PEG@cNGR probe thus makes it prospective for accessing the metastasis and malignancy of the tumor, even for assessing the effect of anti-angiogenic agents. In addition, the obtained GNPs with the optimized sizes of about 13 nm could be applied as X-ray theranostic agents for simultaneous enhanced CT imaging and radiosensitization.^21^ Therefore, the obtained GNPs–PEG@cNGR probe is expected to serve as a radiosensitizer used for tumor radiotherapy. We firmly believe that our synthetic method for optimizing cNGR-functionalized gold nanoparticles can be further extended to fabricate other target ligands directly through the Au–S bonds. Thus, the clinical translation of the GNPs–PEG@cNGR as a tumor angiogenesis imaging agent could be significantly meaningful.

## Conflicts of interest

There are no conflicts to declare.

## Supplementary Material

RA-008-C7RA10155D-s001
