# Supplementary material for: Tumor angiogenesis targeting and imaging using gold nanoparticle probe with directly conjugated cyclic NGR
Source: RSC Adv. 2018 Jan 5;8(3):1706–16. doi: 10.1039/c7ra10155d (PMC9077115; doi:10.1039/c7ra10155d)
Supplement: RA-008-C7RA10155D-s001 [file RA-008-C7RA10155D-s001.pdf]

Electronic Supplementary Material (ESI) for RSC Advances.
This journal is © The Royal Society of Chemistry 2018

Electronic supplementary information (ESI)

Tumor Angiogenesis Targeting and CT Imaging Using Gold Nanoparticle Probe with Directly Conjugated cyclic NGR

Minghao Wu[#a], Yanyan Zhang[#a], Ying Zhang[a], Mingjie Wu[b], Menglin Wu[a], Hongyi Wu[a], Lin Cao[a], Liang Li[a], Xue Li[*a], Xuening Zhang[*a]

[a] *Department of Medical Imaging, Second Hospital of Tianjin Medical University, Tianjin 300211, P. R. China*

[b] *Institut National de la Recherche Scientifique-Énergie Matériaux et Télécommunications, Varennes, Quebec, Canada J3X 1S2*

[#] *Authors who contributed equally to this work.*

Corresponding authors:

*Xuening Zhang, Tel:022-88329407. E-mail: luckyxn_tianjin@163.com

*Xue Li, Tel:022-88329407. E-mail: lx_1229@163.com

Figure S1 UV-visble absorption spectrum of the GNPs-PEG@cNGRs probe dispersed into PBS (A) and cell culture medium DMEM (B) with the increase of storage time for 120 days. Inset: pictures of the probes under different solvent conditions kept in 1.5 ml EP tube for 120 days.

Figure S2 Hydrodynamic size distribution of the GNPs-PEG@cNGRs incubated with PBS (C) and cell culture medium DMEM (D) at 37 ^{o}C for 0 and 120 days.

Figure S3 MTT viability assay of 293T cell after treatment with GNPs-PEG@cNGR at different concentration from 18.75 to 300 μg/mL for 24 h.

Figure S4 Biodistribution of the probe GNPs-PEG@cNGR (A) and GNPs-PEG (B) at 1, 2, 4, 8, and 24 h post injection.

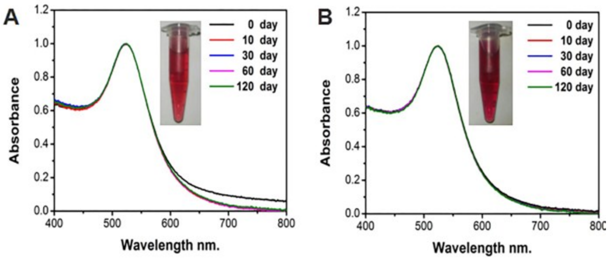

Figure S1

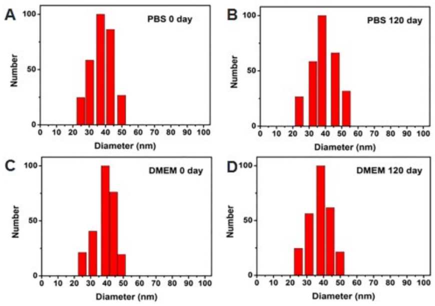
A
PBS 0 day
Number
Diameter (nm)
B
PBS 120 day
C
DMEM 0 day
D
DMEM 120 day

Figure S2

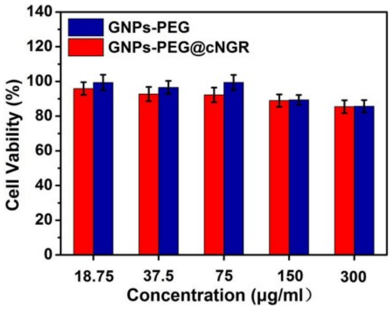
GNPs-PEG
GNPs-PEG@cNGR
Cell Vability (%)
140
120
100
80
60
40
20
0
18.75
37.5
75
150
300
Concentration (μg/ml）

Figure S3

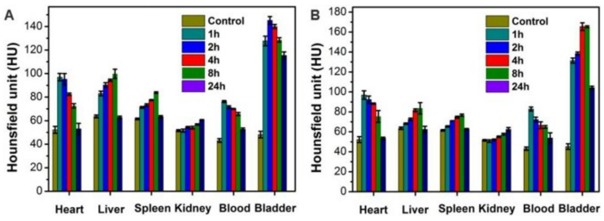
A
Control
1h
2h
4h
8h
24h
Hounsfield unit (HU)
0
20
40
60
80
100
120
140
Heart
Liver
Spleen
Kidney
Blood
Bladder
B
Control
1h
2h
4h
8h
24h
Hounsfield unit (HU)
0
20
40
60
80
100
120
140
160
180
Heart
Liver
Spleen
Kidney
Blood
Bladder

Figure S4
